# White Matter Hyperintensities Modify *CYP2C19* Effect on Stroke Progression in Small Vessel Occlusion

**DOI:** 10.1002/acn3.70579

**Published:** 2026-11-13

**Authors:** Xiaoxin Sun, Xinxiu Liu, Lei Wang, Rui Zhu, Miaomiao Wang, Zhiyong Zhang

**Affiliations:** ^1^ Department of Neurology Beijing Geriatric Hospital (Capital Medical University Geriatric Teaching Hospital) Beijing China; ^2^ Neurovascular Intervention Center Beijing Geriatric Hospital (Capital Medical University Geriatric Teaching Hospital) Beijing China; ^3^ Neuromedicine Center Beijing Geriatric Hospital (Capital Medical University Geriatric Teaching Hospital) Beijing China

**Keywords:** cerebral small vessel disease, lacunar, leukoaraiosis, loss of function mutation, stroke

## Abstract

**Background:**

The association between *CYP2C19* loss‐of‐function (LOF) alleles and early neurological deterioration (END) in acute small vessel occlusion (SVO) patients receiving dual antiplatelet therapy (DAPT) remains inconsistent. We hypothesized that white matter hyperintensities (WMH) burden act as a key moderator and investigated whether this modulates the association between *CYP2C19* LOF and END.

**Methods:**

We conducted a retrospective study of SVO patients receiving DAPT. *CYP2C19* genotypes were classified as LOF carriers or non‐carriers. The primary endpoint was END; secondary and safety endpoints included severe END, 90‐day mRS 0–2, symptomatic intracranial hemorrhage (sICH), bleeding, and mortality. Multivariable regression and interaction analyzes assessed associations between *CYP2C19*, WMH, and clinical outcomes.

**Results:**

A total of 247 patients (median age 67.0 years; 28.7% female; 60.7% *CYP2C19* LOF carriers), 54 (21.9%) experienced END. Multivariable analysis identified infarct diameter (aOR 1.26, 95% CI 1.13–1.39, *p* < 0.001) and total cerebral small vessel disease (CSVD) burden (aOR 2.02, 95% CI 1.37–2.98, *p* < 0.001) as independent predictors. A significant multiplicative interaction was observed between *CYP2C19* LOF and severe WMH on END risk (aOR 9.34, 95% CI 2.06–48.10, *p* = 0.005). Stratified analysis showed *CYP2C19* LOF significantly increased END risk specifically in patients with severe WMH (aOR 3.44, 95% CI 1.12–10.55, *p* = 0.030), but not mild WMH. Predicted probability curves suggested a Fazekas score threshold of approximately 2.70. Secondary and safety outcomes were infrequent.

**Conclusions:**

*CYP2C19* LOF alleles increase END risk in SVO patients specifically with severe WMH. These findings highlight the value of integrating genetic and neuroimaging markers for risk stratification and personalized antiplatelet therapy.

## 1 Introduction

Small vessel occlusion (SVO) accounts for approximately 25% of all ischemic strokes [1]. Although SVO typically presents with mild initial deficits, early neurological deterioration (END) occurs in 16.9%–30.0% of patients and frequently leads to poor long‐term outcomes [2]. Despite guideline‐recommended dual antiplatelet therapy (DAPT) [3], END still occurs in a subset of patients, and targeted prevention remains challenging. The mechanisms of END are multifactorial, with thrombus extension driven by platelet activation as a key contributor [4].

Previous studies have identified multiple risk factors for END in SVO, including infarct location and size, baseline National Institutes of Health Stroke Scale (NIHSS) score, and capsular warning syndrome (CWS) [2, 5, 6, 7, 8]. Clopidogrel resistance is an important driver of this residual risk. As a prodrug, clopidogrel requires hepatic *CYP2C19* metabolism to produce its active metabolite. Loss‐of‐function (LOF) alleles, predominantly CYP2C19*2 and *3, are prevalent in approximately 60% of East Asian populations, reducing active metabolite generation and platelet inhibition [9]. Although LOF carriers face an approximately doubled risk of END [4, 10], *CYP2C19* genotype alone explains only 10%–12% of clinical response variability, suggesting that other physiological or genetic factors interact with LOF alleles to modulate antiplatelet efficacy [11].

White matter hyperintensities (WMH) are the neuroimaging hallmark of chronic cerebral small vessel disease (CSVD), reflecting cumulative microvascular damage and endothelial dysfunction [8, 12]. Because WMH and SVO share underlying small‐vessel pathology [13], a severe WMH burden reflects reduced baseline cerebrovascular reserve [14]. When acute SVO occurs, such pre‐existing vascular compromise leaves the tissue highly vulnerable to perfusion failure, contributing to an increased risk of END [15, 16].

We hypothesized that *CYP2C19* LOF carrier status and severe WMH burden interact to increase the risk of END, as insufficient platelet inhibition may lead to critical perfusion failure in an already compromised microvascular bed. Whether WMH burden modifies the effect of *CYP2C19* LOF on END remains unexplored. Therefore, this retrospective cohort study investigated this gene‐imaging interaction in SVO patients receiving DAPT, aiming to provide a basis for more precise risk stratification and personalized antiplatelet strategies.

## 2 Methods

### 2.1 Study Design and Populations

This single‐center, retrospective observational study was approved by the Ethics Committee of Beijing Geriatric Hospital (approval No. 2023‐030). The requirement for informed consent was waived due to the retrospective nature of the analysis. The study was conducted in accordance with the Declaration of Helsinki.

Between January 2023 and December 2025, 1208 acute ischemic stroke patients were admitted to the Department of Neurology, Beijing Geriatric Hospital. Inclusion criteria were: (1) admission within 48 h of onset; (2) age ≥ 18 years; (3) MRI‐confirmed diagnosis of SVO; (4) availability of complete brain MRI data for CSVD burden assessment; (5) treatment with aspirin plus clopidogrel; and (6) availability of *CYP2C19* genotyping. Exclusion criteria included: (1) other determined stroke etiologies; (2) contraindications to DAPT (active bleeding, severe hepatic or renal impairment, platelet count < 100 × 10^9^/L); (3) pre‐stroke modified Rankin Scale (mRS) score > 2; (4) received intravenous thrombolysis or endovascular therapy; and (5) lack of baseline data. Finally, 247 patients were eligible for the analysis (Figure 1). Follow‐up mRS assessment at 90 days was completed in 240 patients (97.2%).

**FIGURE 1:**
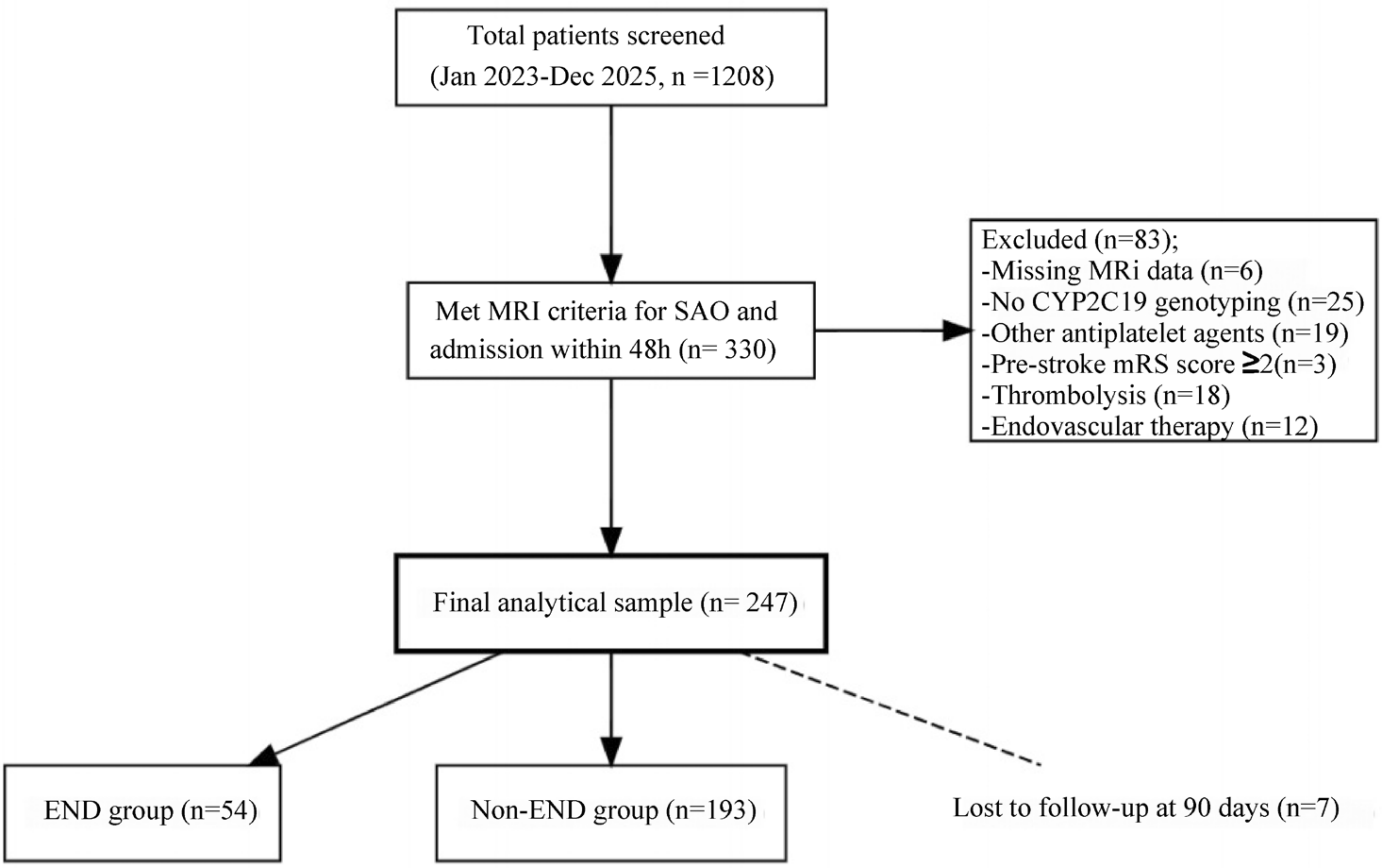
Study flowchart. A total of 1208 patients with acute ischemic stroke were screened. After excluding 961 cases, 247 SVO patients receiving DAPT were included. Of these, 54 (21.9%) developed END. Follow‐up at 90 days was completed in 240 patients (97.2%), with 7 lost to follow‐up. END, early neurological deterioration; DAPT, dual antiplatelet therapy; SVO, small vessel occlusion.

Stroke subtypes were classified according to the Trial of Org 10,172 in Acute Stroke Treatment (TOAST) criteria [17]. For the SVO subtype, we adopted a modified definition based on previously established criteria: a single acute infarct within the territory of a penetrating artery, with a maximum axial diameter of < 20 mm on DWI, located in the subcortical area or brainstem, and without evidence of > 50% stenosis in the parent artery or a cardioembolic source [18].

### 2.2 Baseline Data Collection

Clinical data were extracted from standardized medical records. Baseline characteristics included demographics, vascular risk factors (hypertension, diabetes mellitus, hyperlipidemia, stroke history, coronary heart disease, atrial fibrillation, smoking, and alcohol use), and admission blood pressure. Neurological status was assessed using the NIHSS at admission and monitored daily, with additional evaluation performed upon suspected clinical deterioration. Functional outcome was assessed at 90 days post‐stroke using the mRS score. Laboratory tests included fasting plasma glucose, hemoglobin A1c, total cholesterol, triglycerides, low‐density lipoprotein cholesterol, high‐density lipoprotein cholesterol, and platelet count. All patients were managed in the stroke unit according to institutional protocols for secondary stroke prevention, including standardized anti‐hypertensive and statin therapies.

### 2.3 Radiological Assessment

Brain MRI was performed using a 3.0 T scanner (Ingenia 3.0 T, Philips, The Netherlands). The imaging protocol included diffusion‐weighted MRI (DWI), T1‐weighted, T2‐weighted, fluid‐attenuated inversion recovery (FLAIR), and susceptibility‐weighted imaging (SWI) sequences. CSVD markers were defined according to the Standards for Reporting Vascular Changes on Neuroimaging (STRIVE) consensus [19]. All images were evaluated by two experienced neuroradiologists blinded to the clinical data, with a third senior neuroradiologist adjudicating any discordant findings.

WMH was evaluated on FLAIR sequences using the Fazekas scale [20, 21], which independently scores periventricular (PVWMH) and deep (DWMH) WMH. The total Fazekas score (range 0–6) was calculated as the sum of PVWMH and DWMH scores. To categorize overall burden, patients were stratified into mild (0–2) and severe (3–6) WMH groups [22, 23]. The total CSVD burden score (range 0–4) was determined based on the method proposed by Klarenbeek et al. [24], assigning one point for each of the following: (1) presence of ≥ 1 lacune; (2) extensive WMH (DWMH Fazekas score ≥ 2 or PVWMH Fazekas score = 3); (3) presence of ≥ 1 cerebral microbleed on SWI; and (4) moderate‐to‐severe basal ganglia enlarged perivascular spaces (score ≥ 2). Acute infarcts were characterized by location as strategic (involving the internal capsule, pons, or corona radiata) or non‐strategic [8, 25], because these strategic regions are rich in corticospinal tract fibers and prone to END.

### 2.4 Genotyping

*CYP2C19* genotypes (*2, *3, and *17) were determined using the GMEX point‐of‐care genotyping system (Chongqing Jingyin Bioscience Co. Ltd.), a platform validated for identifying polymorphisms from buccal swabs and utilized in the CHANCE‐2 trial. Based on the genotype, patients were classified into LOF carriers and non‐carriers. LOF carriers were defined as individuals carrying at least one LOF allele (*2 or *3), including genotypes such as *2/*2, *3/*3, *2/*3, *1/*2, *1/*3, *2/*17, and *3/*17. Non‐carriers were defined as individuals without LOF alleles, including normal metabolizer (*1/*1) and rapid/ultrarapid metabolizer (*1/*17 or *17/*17).

### 2.5 Outcome Measurement

Clinical outcomes were assessed through a combination of electronic medical records review and systematic telephone follow‐up. The primary endpoint was END, defined as an increase of ≥ 2 points in total NIHSS score or ≥ 1 point in motor items within 72 h of admission [26, 27]. Secondary endpoints included severe END (NIHSS increase ≥ 4 points within 7 days) [28] and favorable functional outcome (mRS 0–2 at 90 days). Safety outcomes included symptomatic intracranial hemorrhage (sICH), any bleeding event, and all‐cause mortality during the 90‐day follow‐up period. sICH was defined by the European Cooperative Acute Stroke Study (ECASS) III criteria as any intracranial hemorrhage on follow‐up imaging (CT or MRI) accompanied by neurological deterioration (≥ 4 points on the NIHSS) [29]. Any bleeding event was defined as a bleeding episode requiring clinical intervention or treatment modification. All outcomes were independently adjudicated by two senior neurologists blinded to the clinical and genetic data.

### 2.6 Statistical Analyses

Continuous variables were presented as mean ± SD or median (IQR) and were compared using Student's *t*‐test or Mann–Whitney *U*‐test. Categorical variables were expressed as frequencies (percentages) and compared by chi‐square or Fisher's exact test. Multivariable logistic regression was used to assess association of WMH burden and *CYP2C19* genotypes with END. Three models were fitted: Model 1 (unadjusted); Model 2 (adjusted for age, sex, baseline NIHSS); Model 3 (further adjusted for fasting blood glucose, admission systolic blood pressure [SBP], smoking, alcohol use, infarct location, and infarct diameter). Multiplicative interaction was tested by including a cross‐product term (Wald test) and confirmed by likelihood ratio test (LRT). Additive interaction was assessed using relative excess risk due to interaction (RERI), attributable proportion (AP), and synergy index (SI) as described by Andersson et al. [30]. Stratified analyzes examined the association between *CYP2C19* LOF carrier status and END across WMH severity subgroups. All analyzes were conducted using SPSS (version 26.0) and R (version 4.5.3). A two‐tailed *p* < 0.05 was considered statistically significant.

## 3 Results

### 3.1 Baseline Characteristics

Baseline characteristics of the 247 patients are summarized in Table S1. The cohort had a median age of 67 (IQR 61–73) years, with 28.7% (*n* = 71) being female. *CYP2C19* LOF carriers accounted for 60.7% (*n* = 150) of the population. During the hospital stay, END occurred in 54 (21.9%) patients, of whom 50 (92.6%) underwent treatment escalation (tirofiban or ticagrelor substituted for clopidogrel per institutional protocol).

In univariate analysis, patients with END had significantly higher admission NIHSS scores (median 2.9 vs. 2.0; *p* < 0.001), larger infarct diameters (median 10.1 vs. 7.0 mm; *p* < 0.001), and higher CSVD total burden scores (median 1.8 vs. 1.3; *p* = 0.005). Infarct location was significantly associated with END (*p* = 0.020), with a higher prevalence of strategic lesions in the END group (75.9% vs. 58.5%). While the proportions of severe WMH and *CYP2C19* LOF carriers were numerically higher in the END group (57.4% vs. 50.3% and 66.7% vs. 59.1%, respectively), these differences did not reach statistical significance (*p =* 0.353 and *p* = 0.312). Admission SBP was higher in the END group than in the non‐END group (161.3 ± 20.9 vs. 156.0 ± 19.6 mmHg), but the difference was not statistically significant (*p* = 0.090). No significant differences were observed between the two groups in age, sex, vascular risk factors, or laboratory parameters.

Multivariable logistic regression (Table 1) showed that, after adjustment for age, sex, SBP, NIHSS, smoking, alcohol, FPG, infarct location, and diameter, infarct diameter emerged as an independent predictor of END (adjusted odds ratio [aOR] 1.26, 95% confidence interval (CI) 1.13–1.39, *p* < 0.001). Conversely, severe WMH (aOR 1.72, 95% CI 0.80–3.70, *p* = 0.167) and CYP2*C19* LOF carrier status (aOR 1.22, 95% CI 0.59–2.54, *p* = 0.591) were not significantly associated with END. In sensitivity analysis using CSVD total score instead of severe WMH (Table S2), a higher CSVD total score was independently associated with END (aOR 2.02, 95% CI 1.37–2.98, *p <* 0.001), while the association between infarct diameter and END remained consistent.

**TABLE 1:** Multivariable logistic regression analysis of factors associated with END.

Variable	Adjusted OR (95% CI)	*p*
Age	0.98 (0.94–1.02)	0.297
Female	1.33 (0.60–2.98)	0.484
SBP	1.01 (0.99–1.02)	0.475
NIHSS	1.02 (0.89–1.19)	0.752
Current smoking	0.80 (0.38–1.69)	0.559
Alcohol consumption	0.63 (0.25–1.58)	0.324
FPG	1.03 (0.90–1.18)	0.659
Strategic locations	1.96 (0.90–4.26)	0.091
Infarct diameter	1.26 (1.13–1.39)	**< 0.001**
Severe WMH	1.72 (0.80–3.70)	0.167
*CYP2C19* LOF carrier	1.22 (0.59–2.54)	0.591

*Note:* Bold values indicate statistical significance (*p* < 0.05).

Abbreviations: CI, confidence interval; END, early neurological deterioration; FPG, fasting plasma glucose; LOF, loss‐of‐function; NIHSS, National Institutes of Health Stroke Scale; OR, odds ratio; SBP, systolic blood pressure; WMH, white matter hyperintensities.

### 3.2 Interactive Effect of CYP2C19 LOF Status and WMH Burden on END

We evaluated the multiplicative interaction between *CYP2C19* LOF carrier status and severe WMH on the risk of END (Table 2). In the fully adjusted model (Model 3), the interaction term was significant (aOR 9.34, 95% CI 2.06–48.10, interaction *p* = 0.005), indicating that the association between LOF status and END is modified by WMH severity. This result was robust upon LRT (interaction *p* = 0.0034). For additive interaction, the relative excess risk due to interaction (RERI) was 1.65 (95% CI 0.57–2.72) and the attributable proportion (AP) was 1.15 (95% CI 0.50–1.80). The synergy index (SI) was not computed as the individual main effects of both LOF and severe WMH showed protective trends (OR < 1) in our model. Despite wide 95% CIs for the interaction terms—likely reflecting the limited number of END events—the direction and statistical significance of the interaction remained consistent across all adjustment models.

**TABLE 2:** Multivariable logistic regression models for the multiplicative interaction between LOF and WMH on END.

Variables	Model 1	Model 2	Model 3
LOF carrier	0.63 (0.25–1.57)	0.65 (0.25–1.66)	0.37 (0.12–1.09)
Severe WMH	0.49 (0.16–1.39)	0.55 (0.17–1.71)	0.41 (0.11–1.44)
LOF carrier × Severe WMH	4.66 (1.26–18.67)	5.36 (1.39–22.49)	9.34 (2.06–48.10)
Interaction *p*	0.024	0.017	0.005

*Note:* Model 1: Unadjusted model including LOF carrier, WMH, and their interaction term. Model 2 (Adjusted): Adjusted for age, sex, and baseline NIHSS score. Model 3 (Fully Adjusted): Adjusted for age, sex, baseline NIHSS score, fasting blood glucose, admission SBP, smoking status, alcohol use, infarct location, and infarct diameter. Interaction *p* were derived from the Wald test; likelihood ratio test results are reported in the text.

Abbreviations: END, early neurological deterioration; LOF, loss‐of‐function; WMH, white matter hyperintensities.

To further characterize this interaction, we performed stratified analysis by WMH burden (Figure 2). The association between *CYP2C19* LOF carrier status and END differed by WMH severity: among patients with mild WMH, LOF status was not significantly associated with END (aOR 0.37, 95% CI 0.12–1.10, *p* = 0.074), whereas among those with severe WMH, LOF status was significantly associated with an increased risk of END (aOR 3.44, 95% CI 1.12–10.55, *p* = 0.030).

**FIGURE 2:**
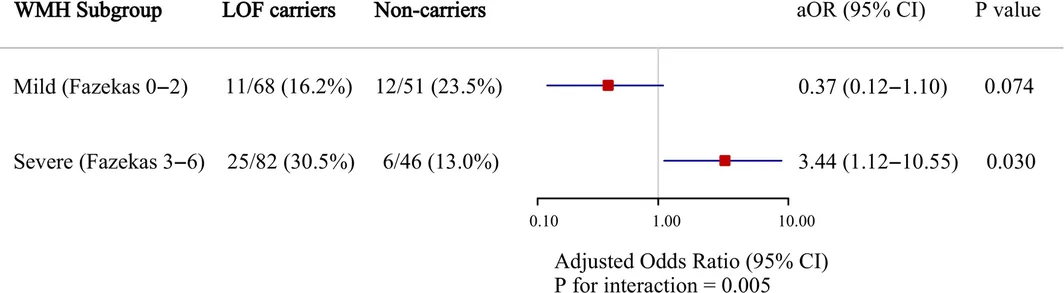
Forest plot of stratified analysis according to WMH burden. For each subgroup, the table shows *CYP2C19* LOF and non‐LOF carrier event rates (*n*/*N*, %), aOR (95% CI), and *p* value. Dashed line at OR = 1. *p* for interaction = 0.005 (multiplicative). aORs and 95% CIs were calculated using multivariable logistic regression models, adjusting for age, sex, baseline NIHSS score, FPG, admission SBP, smoking status, alcohol use, infarct location, and infarct diameter. aOR, adjusted odds ratio; CI, confidence interval; END, early neurological deterioration; FPG, fasting plasma glucose; LOF, loss‐of‐function; NIHSS, National Institutes of Health Stroke Scale; SBP, systolic blood pressure; WMH, white matter hyperintensities.

### 3.3 Continuous WMH Analysis and Threshold Effect

When analyzing the Fazekas score as a continuous variable, the interaction term (LOF × Fazekas) was not significant (aOR 1.45, 95% CI 0.87–2.43, *p* = 0.156). However, predicted probability curves (Figure 3) intersected at a Fazekas score of approximately 2.70; beyond this point, the predicted risk of END among LOF carriers shifted from lower to higher relative to non‐carriers. This intersection, combined with the significant multiplicative interaction observed with the binary WMH classification (Fazekas ≥ 3), suggests a potential threshold effect of WMH burden on the clinical impact of *CYP2C19* LOF variants.

**FIGURE 3:**
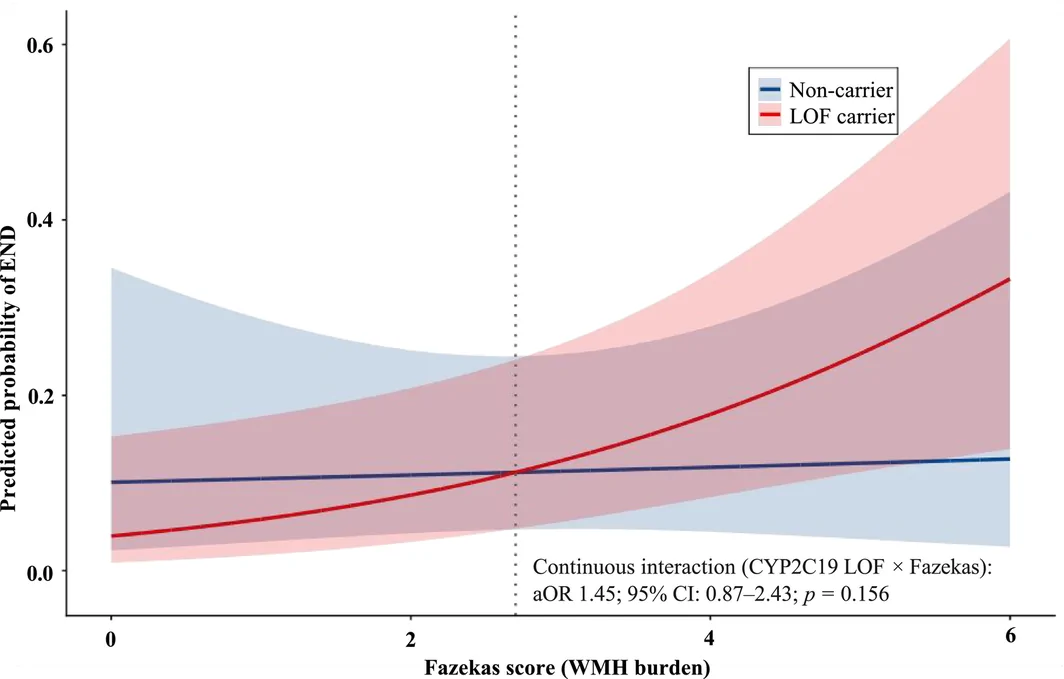
Interaction between *CYP2C19* LOF carrier status and Fazekas score (continuous) on the predicted probability of END. The predicted probability of END increases more sharply in LOF carriers compared to non‐carriers as the Fazekas score rises. Despite a non‐significant continuous interaction term (*p* = 0.156), the curves intersect at a Fazekas score of approximately 2.70, suggesting that the clinical impact of LOF variants may be exacerbated by severe WMH burden. Shaded areas represent 95% CIs. aOR, adjusted odds ratio; CI, confidence interval; END, early neurological deterioration; LOF, loss‐of‐function; WMH, white matter hyperintensities.

### 3.4 Secondary and Safety Outcomes

Secondary and safety outcomes are summarized in Table 3. Severe END occurred in 6.7% (10/150) of LOF carriers and 5.2% (5/97) in non‐carriers. At 90 days, a favorable functional outcome (mRS 0–2) was achieved by 95.2% (140/147) of LOF carriers and 97.8% (91/93) of non‐carriers. Safety events were infrequent: sICH occurred in only 3 patients (1.2%; 1 LOF carrier and 2 non‐carriers), and any bleeding event was reported in 1 (0.7%) LOF carrier. No mortality was recorded during the 90‐day follow‐up. Formal statistical comparisons were not conducted due to the low frequency of these clinical events.

**TABLE 3:** Primary and secondary outcomes.

Study endpoints	LOF carriers (*n* = 150)	Non‐carriers (*n* = 97)
Primary outcome, *n* (%)		
END	36 (24.0)	18 (18.6)
Secondary outcomes, *n* (%)		
Severe END	10 (6.7)	5 (5.2)
mRS score of 0 to 2 at 90 days^a^	140 (95.2)	91 (97.8)
Safety outcomes, *n* (%)^a^		
sICH	1 (0.7)	2 (2.2)
Any bleeding event	1 (0.7)	0 (0.0)
All‐cause mortality within 90 days	0 (0.0)	0 (0.0)

Abbreviations: END, early neurological deterioration; LOF, loss‐of‐function; mRS, modified Rankin Scale; sICH, symptomatic intracranial hemorrhage.

^a^ Percentages were calculated among patients who completed the 90‐day follow‐up (147 LOF carriers and 93 non‐carriers). Due to the low event counts, no formal statistical comparisons were performed.

## 4 Discussion

In this retrospective cohort of 247 patients with SVO, the incidence of END was 21.9%. Larger infarct diameter and higher CSVD total burden independently predicted END. We identified a significant multiplicative interaction between *CYP2C19* LOF alleles and severe WMH burden on END risk. While LOF status alone did not independently predict END, it increased the risk over threefold in patients with severe WMH but not in those with mild WMH. These findings suggest that the clinical expression of clopidogrel resistance may depend on the degree of pre‐existing microvascular damage. This novel gene‐imaging interaction may guide targeted early intensified antiplatelet therapy in high‐risk LOF carriers.

The underlying mechanism likely involves a “two‐hit” model of END in SVO. Severe WMH reflects chronic microvascular damage, endothelial dysfunction, and blood–brain barrier disruption [16, 31], with impaired hemodynamic regulation [32], which together lead to exhausted microcirculatory reserve, impaired autoregulation, and compromised collateral flow [14, 33, 34, 35]. Zerna et al. found that WMH burden increased short‐term disability risk mainly when intracranial stenosis or occlusion was present, supporting WMH as an effect modifier [36]. In this vulnerable state, suboptimal platelet inhibition due to *CYP2C19* LOF may act as a second hit, such that even minor thrombus propagation could precipitate END [4]. Conversely, mild WMH retains sufficient reserve to buffer this effect, which may explain why the genetic risk is only observed beyond a certain threshold.

Our observed LOF carrier frequency (60.7%) aligns with East Asian demographics [37, 38]. Prior meta‐analyzes linking LOF alleles to poor outcomes (RR 1.63) often included heterogeneous populations such as large artery atherosclerosis and cardioembolic strokes, where thrombotic burden predominates; however, this association was less consistent in SVO [39]. In our homogeneous SVO cohort with uniform DAPT, the lack of independent predictive value for LOF status suggests that the clinical impact of clopidogrel resistance may depend on stroke subtype and underlying pathophysiology.

WMH burden significantly modified the effect of *CYP2C19* LOF status, with LOF‐associated risk mainly in severe WMH (Fazekas ≥ 3). This threshold pattern is consistent with prior studies: Ryu et al. found that only the highest WMH quintile predicted 3‐month outcomes in SVO [15], and Guo et al. linked severe leukoaraiosis to increased END risk after thrombectomy [40]. Although the continuous Fazekas score × LOF interaction was not significant, the probability curves cross at a Fazekas score of approximately 2.70, suggesting a threshold effect; however, this cutoff should be interpreted cautiously given the limited sample at the score extremes.

The CSVD total burden score remained a robust predictor of END, likely because it integrates a broader range of small vessel pathology—lacunes, microbleeds, and enlarged perivascular spaces—beyond WMH alone [41]; these lesion types may contribute to END via distinct mechanisms.

Regarding infarct morphology, larger infarct diameter was independently associated with END (aOR 1.26 per 1 mm increase), consistent with Huang et al. [42], who reported a sharp rise in END risk at diameter > 15 mm. Infarcts > 15 mm are more characteristic of branch atheromatous disease (BAD) than classic lipohyalinotic SVO, explaining their higher susceptibility to progression [43]. While our SVO criteria (lesion diameter < 20 mm) align with established standards, this threshold may introduce pathophysiological heterogeneity. Future studies using high‐resolution vessel wall imaging are needed to refine this definition. Additionally, previous studies have identified CWS as a strong predictor of END (aOR 7.00) [8]. However, CWS was not systematically recorded in our retrospective cohort, and internal capsule involvement was comparable between groups (7.4% vs. 6.7%). Because CWS is a clinical syndrome rather than an imaging finding, these data cannot exclude it as a potential unmeasured confounder.

Finally, 90‐day functional recovery was generally favorable, consistent with previous DAPT trials in SVO [44]. sICH was rare, and no moderate or severe bleeding events occurred. Given the limited sample size and the treatment heterogeneity, we did not perform formal statistical comparisons for safety endpoints. Nonetheless, the absence of excess bleeding in patients receiving intensified therapy suggests that genotype‐guided antiplatelet adjustment is safe even with severe WMH burden, consistent with the INSPIRES trial, where WMH severity did not modify bleeding risk [45].

### 4.1 Limitations

The present study has several limitations. First, its retrospective, single‐center design within a Chinese population and relatively small sample size may limit generalizability and statistical power; thus, our findings require validation in larger, multicenter prospective cohorts. Second, non‐standardized treatment escalation and the low frequency of bleeding events did not allow for a direct comparison across strategies. Although the low bleeding rate suggests that individualized therapy for LOF carriers is well tolerated, these findings should be interpreted with caution given the small sample and treatment heterogeneity. Third, we used the visual Fazekas scale rather than volumetric quantification. While volumetric analysis is more precise, the Fazekas scale is highly correlated with quantitative measures and is more feasible for daily clinical routine. Fourth, our SVO criteria (lesion diameter < 20 mm) likely included patients with BAD. While this reflects real‐world clinical heterogeneity, future studies incorporating high‐resolution vessel wall imaging are needed to better distinguish these pathological entities. Fifth, our study focused solely on major *CYP2C19* genotypes (*2, *3, and *17) without screening for other variants (e.g., *P2Y12* and *GPIIIa*) or performing platelet function testing, potentially failing to capture gene–gene interactions and the full pharmacodynamic response to clopidogrel [4]. Sixth, because of the retrospective design, clinical fluctuations typical of CWS were not systematically recorded, representing a potential unmeasured confounder. Additionally, infarct location was simplified into strategic versus non‐strategic categories to avoid model overfitting, which may have prevented more detailed analyzes of specific subregions. Finally, we could not include all potential confounders in our multivariable models, and residual or unmeasured confounding may remain and could partly explain the observed associations. Furthermore, excluding patients unable to complete MRI may have introduced selection bias toward less severe cases, potentially underestimating total event rates.

## 5 Conclusions

In conclusion, the impact of *CYP2C19* LOF alleles on END in acute SVO patients was significantly modulated by WMH burden. *CYP2C19* status was associated with END specifically in patients with severe WMH, suggesting that structural brain vulnerability amplifies the clinical consequences of suboptimal platelet inhibition. These findings underscore that genotype should not be interpreted in isolation and that integrating genetic testing with neuroimaging assessment may refine risk stratification for personalized antiplatelet therapy. Future studies are needed to validate this gene–imaging interaction and explore whether alternative P2Y12 inhibitors provide superior clinical benefits in this high‐risk subgroup.

## Author Contributions

X.S. and Z.Z. had full access to all the data in the study and took responsibility for the integrity of the data and the accuracy of the data analysis. Concept and design: X.S. and Z.Z. Acquisition, analysis or interpretation of data: X.S., X.L., L.W., R.Z., M.W., and Z.Z. Drafting of the manuscript: X.S. Critical revision of the manuscript for important intellectual content: X.S., X.L., L.W., R.Z., M.W., and Z.Z. Statistical analysis: X.S. and Z.Z. Obtained funding: Z.Z. Administrative, technical or material support: X.L., L.W., R.Z., and M.W. Supervision: Z.Z.

## Funding

This work was supported by the Research Funds of Beijing Geriatric Hospital (Grant No. 2022bjlnyy‐xh‐3) and the Beijing Geriatric Hospital Key Specialty/Discipline Construction Fund.

## Ethics Statement

The study was conducted in accordance with the Declaration of Helsinki and was approved by the Ethics Committee of Beijing Geriatric Hospital (approval No. BJLNYY‐2023‐030). The requirement for informed consent was waived due to the retrospective nature of the analysis.

## Consent

The authors have nothing to report.

## Conflicts of Interest

The authors declare no conflicts of interest.

## Supporting information


**Table S1:** Baseline Characteristics of patients by END status.

**Table S2:** Multivariable logistic regression analysis of factors associated with END.

## Data Availability

The data that support the findings of this study are available from the corresponding author upon reasonable request.
