# Supplementary material for: White Matter Hyperintensities Modify CYP2C19 Effect on Stroke Progression in Small Vessel Occlusion
Source: Ann Clin Transl Neurol. 2026 Nov 13:10.1002/acn3.70579. Online ahead of print. doi: 10.1002/acn3.70579 (PMC13768562; doi:10.1002/acn3.70579)
Supplement: Supplementary file 1 [file ACN3-9999-0-s001.doc]

| **Table S1. Baseline Characteristics of patients by END status** | | | | |
| --- | --- | --- | --- | --- |
| **Characteristics** | **Total**  **(N=247)** | **END group**  **(n=54)** | **Non-END group**  **(n=193)** | **P value** |
| Age, years, median (IQR) | 67 (61, 73) | 65 (59, 73) | 68 (62, 74) | 0.106 |
| Female, n (%) | 71 (28.7) | 18 (33.3) | 53 (27.5) | 0.399 |
| SBP, mmHg, mean ± SD | 157.2 ± 20.0 | 161.3 ± 20.9 | 156.0 ± 19.6 | 0.090 |
| DBP, mmHg, mean ± SD | 88.5 ± 14.1 | 91.1 ± 12.9 | 87.7 ± 14.3 | 0.114 |
| NIHSS, median (IQR) | 2.2 (1.0, 3.0) | 2.9 (1.0, 4.0) | 2.0 (0.0, 3.0) | **<0.001** |
| **Medical history**^*^**, n (%)** |  |  |  |  |
| Hypertension | 210 (85.0) | 48 (88.9) | 162 (83.9) | 0.367 |
| Diabetes | 113 (45.7) | 23 (42.6) | 90 (46.6) | 0.598 |
| Dyslipidemia | 169 (68.4) | 32 (59.3) | 137 (71.0) | 0.101 |
| Coronary heart disease | 60 (24.3) | 14 (25.9) | 46 (23.8) | 0.751 |
| Hyperhomocysteinemia | 96 (38.9) | 21 (38.9) | 75 (38.9) | 0.997 |
| Previous ischemic stroke | 33 (13.4) | 8 (14.8) | 25 (13.0) | 0.722 |
| Renal impairment | 21 (8.5) | 5 (9.3) | 16 (8.3) | 0.821 |
| Current smoking | 109 (44.1) | 19 (35.2) | 90 (46.6) | 0.134 |
| Alcohol consumption | 53 (21.5) | 8 (14.8) | 45 (23.3) | 0.179 |
| **Laboratory results, median (IQR)** |  |  |  |  |
| TC, mmol/L | 4.5 (3.7, 5.2) | 4.5 (3.7, 5.1) | 4.5 (3.7, 5.3) | 0.930 |
| TG, mmol/L | 2.0 (1.2, 2.4) | 2.0 (1.2, 2.5) | 1.9 (1.2, 2.4) | 0.591 |
| LDL-C, mmol/L | 3.0 (2.3, 3.9) | 3.0 (2.4, 3.6) | 3.0 (2.2, 3.9) | 0.905 |
| FPG, mmol/L | 6.8 (5.0, 8.1) | 6.8 (4.8, 8.1) | 6.8 (5.0, 7.9) | 0.948 |
| HbA1c, % | 6.7 (5.4, 7.5) | 6.8 (5.6, 7.8) | 6.6 (5.4, 7.5) | 0.670 |
| PLT, ×10^9^/L | 213.7 (179.0, 246.0) | 223.5 (191.5, 263.8) | 211.0 (178.0, 245.5) | 0.111 |
| **Infarct location, n (%)** |  |  |  |  |
| Strategic locations | 154 (62.3) | 41 (75.9) | 113 (58.5) | **0.020** |
| Internal capsule | 17 (6.9) | 4 (7.4) | 13 (6.7) | — |
| Pons | 69 (27.9) | 20 (37.0) | 49 (25.4) | — |
| Corona radiata | 68 (27.5) | 17 (31.5) | 51 (26.4) | — |
| Non-strategic locations | 93 (37.7%) | 13 (24.1) | 80 (41.5) | — |
| **Infarct diameter, mm, median (IQR)** | 7.7 (5.8, 8.3) | 10.1 (6.5, 13.3) | 7.0 (5.6, 7.9) | **<0.001** |
| **CSVD burden** |  |  |  |  |
| CSVD total score, median (IQR) | 1.4 (1.0, 2.0) | 1.8 (1.0, 2.3) | 1.3 (0.0, 2.0) | **0.005** |
| **WMH burden** |  |  |  |  |
| Fazekas score, median (IQR) | 3.0 (2.0, 4.0) | 3.3 (2.0, 5.0) | 2.9 (2.0, 4.0) | 0.102 |
| Severe WMH, n (%) | 128 (51.8) | 31 (57.4) | 97 (50.3) | 0.353 |
| **Genetic** |  |  |  |  |
| CYP2C19 LOF carrier, n (%) | 150 (60.7) | 36 (66.7) | 114 (59.1) | 0.312 |

^*^ Hypertension, diabetes, hyperlipidemia, coronary heart disease, hyperhomocysteinemia, and renal impairment were defined as diagnosed by a medical doctor or use of specific medication. Strategic locations were defined as internal capsule, pons, or corona radiata. SBP, systolic blood pressure; DBP, diastolic blood pressure; NIHSS, National Institutes of Health Stroke Scale; TC, total cholesterol; TG, triglycerides; LDL-C, low-density lipoprotein cholesterol; FPG, fasting plasma glucose; HbA1c, hemoglobin A1c; PLT, platelet count; CSVD, cerebral small vessel disease; WMH, white matter hyperintensities; LOF, loss-of-function; IQR, interquartile range; SD, standard deviation.
