# Supplementary material for: White Matter Hyperintensities Modify CYP2C19 Effect on Stroke Progression in Small Vessel Occlusion
Source: Ann Clin Transl Neurol. 2026 Nov 13:10.1002/acn3.70579. Online ahead of print. doi: 10.1002/acn3.70579 (PMC13768562; doi:10.1002/acn3.70579)
Supplement: Supplementary file 2 [file ACN3-9999-0-s002.doc]

| **Table S2. Multivariable logistic regression analysis of factors associated with END** | | |
| --- | --- | --- |
| **Variable** | **Adjusted OR (95% CI)** | **P value** |
| Age | 0.96 (0.92–1.00) | 0.062 |
| Female | 1.35 (0.59–3.07) | 0.478 |
| SBP | 1.01 (0.99–1.03) | 0.470 |
| NIHSS | 1.00 (0.86–1.16) | 0.974 |
| Current smoking | 0.85 (0.40–1.84) | 0.688 |
| Alcohol consumption | 0.72 (0.27–1.89) | 0.503 |
| FPG | 1.06 (0.93–1.21) | 0.400 |
| Strategic Locations | 1.96 (0.88–4.36) | 0.101 |
| Infarct diameter | 1.28 (1.15–1.44) | **<0.001** |
| CSVD total score | 2.02 (1.37–2.98) | **<0.001** |
| CYP2C19 LOF carrier | 1.17 (0.56–2.49) | 0.676 |

This sensitivity analysis substituted the CSVD total score for severe WMH. Models were adjusted for the same covariates as the primary model in Table 1, with the exception of severe WMH, which was replaced by the CSVD total score.

END, early neurological deterioration; OR, odds ratio; CI, confidence interval; SBP, systolic blood pressure; NIHSS, National Institutes of Health Stroke Scale; FPG, fasting plasma glucose; CSVD, cerebral small vessel disease ; LOF, loss-of-function.
